# Curcumin Increased the Sensitivity of Non-Small-Cell Lung Cancer to Cisplatin through the Endoplasmic Reticulum Stress Pathway

**DOI:** 10.1155/2022/6886366

**Published:** 2022-06-17

**Authors:** Lile Wang, Ruicheng Hu, Aiguo Dai

**Affiliations:** ^1^Department of Respiratory Medicine, Hunan Provincial People's Hospital (The First-affiliated Hospital of Hunan Normal University), Changsha 410016, China; ^2^Department of Respiratory Diseases, Medical School, Hunan University of Chinese Medicine, Changsha 410208, China

## Abstract

**Objective:**

Non-small-cell lung cancer (NSCLC) is one of the most lethal cancers. Although cisplatin-based chemotherapies have been regarded as a promising treatment approach, cisplatin resistance still remains one of the major clinical challenges. Curcumin, a naturally occurring polyphenol, has been proved to increase chemotherapeutic efficiency of NSCLC cells. However, the role of curcumin in cisplatin-resistant NSCLC cells has been rarely investigated. This study aims to investigate whether curcumin enhances cisplatin sensitivity of human NSCLC cells and its underlying mechanisms.

**Method:**

A549/DDP and H1299/DDP cells were treated by DDP or/and curcumin before cell viability, and apoptosis were determined by using a CCK-8 assay and flow cytometer. The expressions of apoptosis and ER stress-related proteins, including cleaved caspase-3, cleaved PARP, CHOP, GRP78, XBP-1, ATF6, and caspase-4, were measured by the qPCR and western blotting. After cotreatment by DDP and curcumin, A549/DDP and H1299/DDP cells were further treated by the ER stress inhibitor, salubrinal (20 *μ*m), after which the cell apoptosis and viability were detected.

**Result:**

Treatment by DDP and curcumin can substantially decrease cell viability, while can increase the cell apoptosis rate, elevate mRNA and protein expressions of apoptosis and ER stress-related proteins, compared with cells treated by DDP or curcumin alone. Salubrinal treatment can counteract the suppressive effect of DDP and curcumin on cell viability and decrease the cell apoptosis of A549/DDP and H1299/DDP cells.

**Conclusion:**

Curcumin can increase the sensitivity of NSCLC to cisplatin through an ER stress pathway and thus can be served as one of the molecular targets for overcoming the cisplatin resistance.

## 1. Introduction

Lung cancer is one of the most lethal cancers worldwide, which can be clinically classified into non-small-cell lung cancer (NSCLC) and small-cell lung cancer (SCLC) [1]. The former accounts for approximately 85% of all lung cancer cases [2, 3]. Platinum agents, including cisplatin (DDP), are considered to be one of the reference drugs for first-line treatment of NSCLC [4]. Although cisplatin-based chemotherapies proved to have a remarkable curative effect on NSCLC patients, cisplatin resistance developed by NSCLC cells continues to be a major challenge in clinic [5, 6]. The strategy of increasing a cisplatin dose can potentially overcome drug resistance but also leads to increased possibility to develop life-threatening adverse side effects. Thus, searching for strategies to increase the cisplatin sensitivity of NSCLC cells will be highly desirable to overcome drug resistance.

The endoplasmic reticulum (ER) is the first intracellular compartment of the secretory pathway, which regulates calcium homeostasis, lipid biosynthesis, and protein productive folding and quality control. However, under certain cytotoxic conditions, such as hypoxia and nutrient deprivation, protein misfolding occurs via disruption of proper ER function, leading to unfolded proteins accumulating and aggregating in the ER, known as ER stress [7–12]. ER stress was associated with anticancer drug resistance via unfolded protein response [10, 13].

Curcumin extracted from the plant of Curcuma longa was reported to possess antitumor activity through regulating oncogene expression, cell cycle regulation, apoptosis, tumorigenesis, and metastasis PMID: 34885693. Recently, curcumin was found to promote chemotherapeutic efficiency in various cancers and diseases [9, 14–17]. For instance, Zou et al. reported that curcumin increases breast cancer cell sensitivity to cisplatin by decreasing FEN1 expression [9]. Lu et al. found that curcumin can increase the sensitivity of paclitaxel-resistant NSCLC cells to paclitaxel through microRNA-30c-mediated MTA1 reduction [16]. So far, the possible effect of curcumin on cisplatin sensitivity of lung cancer cell has not been well investigated and worthy further explorations. Recently, curcumin was found to increase effects of irinotecan through mediating the ER stress pathway in colorectal cancer cells [18, 19]. Misra et al. found that curcumin regulates ER stress through cAMP responsive element-binding protein H [20]. So far, the implication of ER stress in curcumin-attenuated drug resistance of lung cancer cells has been rarely investigated.

Inspired by previous studies, this study intends to investigate whether the ER stress pathway is the key factor for curcumin-increased NSCLC sensitivity to cisplatin, with the expectation to provide a new strategy and molecular target for overcoming the cisplatin resistance in lung cancer cells.

## 2. Materials and Methods

### 2.1. Cell Culture

Human lung cancer cell lines A549 and H1299 originated from the American Type Culture Collection were cultured in the RPMI-1640 medium supplemented with 10% fetal bovine serum (FBS, Gibco) in a humidified atmosphere containing 5% CO_2_ at 37°C. To establish cisplatin-resistant A549 and H1299 cell lines, A549 and H1299 cells were firstly treated with a 0.5 *μ*M of cisplatin (DDP, Sigma) and then were treated with increased concentrations of DDP in a stepwise manner during cell passage. To maintain the drug-resistant phenotype, DDP (with final concentration of 1.5 *μ*M) was added into the culture media for A549/DDP and H1299/DDP cells.

### 2.2. Cell Proliferation Detected by the CCK-8 Assay

A549, A549/DDP, H1299 and, H12999/DDP cells were seeded in 96-well plates at the density of 5 × 10^3^/well. After the cell culture for 12 h, cells were then treated with various concentrations of curcumin (1.25, 2.5, 5, 10, and 20 *μ*g/mL) or/and cisplatin (2, 4, 8, 10, and 12 *μ*g/mL). A549/DDP and H12999/DDP cells were treated by 2 *μ*g/mL DDP, 2.5 *μ*g/mL curcumin or DDP + curcumin, which were, respectively, named as the DDP group, curcumin group, and curcumin + DDP group. Equal volume of PBS was used in the control group. After cell incubation for 48 h, the 10 *μ*L CCK-8 (Sigma-Aldrich, USA) reagent was added to each well for incubation of 2 h. The absorbance at 450 nm in each well was determined by using a microplate reader.

### 2.3. Cell Apoptosis Detected by the Flow Cytometry Assay

The cell apoptosis rate was detected by using the Annexin V-FITC/PI kit (BD Biosciences) based on the instructions. Cells digested by pancreatin were collected after centrifugation, and after that, cells were then resuspended in a binding buffer before further incubation with Annexin V-FITC and PI for 15 min. Cell apoptosis was calculated using a flow cytometry method. All experiments were conducted 3 times.

### 2.4. Protein Expression Levels by the Western Blotting Assay

The expression levels of protein-activating transcription factor 6 (ATF6), C/EBP homology protein (CHOP), and caspase-4 were detected by the western blotting assay. The logarithmic growth cells were inoculated on 6-well plates at the density of 1 × 10^5^ cells/well. Each well was added with corresponding concentrations of curcumin or cisplatin solution in a serum-containing medium. After the cells were lysed, we collected the supernatant and then extracted the cytosolic protein or nuclear protein. The protein centrifugation liquid was transferred into the PVDF membrane after SDS-PAGE vertical electrophoresis. Electrophoretic analysis of equivalent protein lysates was performed.

### 2.5. Real-Time Quantitative PCR Assay Detected Expression of mRNA

Total RNA was isolated from cells using the TRIZOL reagent and then subjected to reverse transcription using the RT kit (TaKaRa, Tokyo, Japan) based on the instructions specified in the kit. The Biosystems 7300 real-time PCR system (ABI, Foster City, CA, USA) was used for PCR using SYBR GreenMix (Takara). Three duplicates were set for each reaction of PCR. Data analysis was determined using the 2^−ΔΔCt^ method [21]: the ^ΔΔ^Ct = experimental group (Ct_target gene_−Ct_internal control_)− the control group (Ct _target gene_−Ct _internal control_). GAPDH was used as the internal control, and primer sequences are listed in Table 1.

### 2.6. Western Blotting

Cells were lysed on ice using RIPA lysis (Beyotime) for 15 min, followed by centrifugation at 13000 g for 5 min. The concentration of proteins was determined using the BCA kit (Beyotime). The proteins were added with a loading buffer for boiling water bath for 10 min. The volume of the loading sample in each group was calculated based on volume of loading proteins. The proteins were treated with electrophoresis at 80 V for 30 min and 120 V for 90 min. The membrane transference was performed at ice water bath at 250 mA for 100 min. Then, the membranes were washed 3 times, each for 1-2 min, and after that, the proteins were incubated with a blocking buffer for 2 h, followed by incubation with following primary antibodies of caspase-3 (ab32351, 1 : 1000, Abcam, UK), cleaved caspase-3 (ab32042, 1 : 1000, Abcam, UK), PARP (ab191217, 1 : 1000, Abcam, UK), cleaved PARP (ab32064, 1 : 1000, Abcam, UK), CHOP (^#^2895S, 1 : 1000, Cell Signaling Technology, CST, Beverly, MA, USA), ATF6 (ab227830, 1 : 1000, Abcam, UK), GRP78 (ab21685, 1 : 1000, Abcam, UK), caspase-4 (ab238114, 1 : 1000, Abcam, UK), and XBP-1 (ab37152, 1 : 1000, Abcam, UK)/GAPDH (ab8245, 1 : 1000, Abcam, UK) at 4°C overnight. The membranes were washed in TBST for 3 times, each for 10 min, and then incubated with horseradish peroxidase labeled goat antirabbit IgG (Beyotime; A0208, 1 : 1000, Shanghai) at room temperature for 2 h, followed by TBST washing 3 times, each for 10 min. The membrane was incubated with the ECL solution (P0018FS, Beyotime, Shanghai) and detected the under color developing system (Bio-Rad). All experiments were performed 3 times.

### 2.7. Statistical Analysis

GraphPad Prism7 was used for data analysis. Experiment data were presented as the mean ± standard deviation (SD). Student's *t*-test was used to analyze statistical significance between pairwise groups, while comparisons among groups were analyzed using one-way analysis of the variance, with Tukey's multiple comparisons for post hoc analysis. Results with *p* < 0.05 and *p* < 0.01 are considered statistically significant (^*∗*^) and (^*∗∗*^), respectively.

## 3. Results

### 3.1. Curcumin Decreases Cell Viability of A549/DDP and H1299/DDP Cells

**Figure 1:**
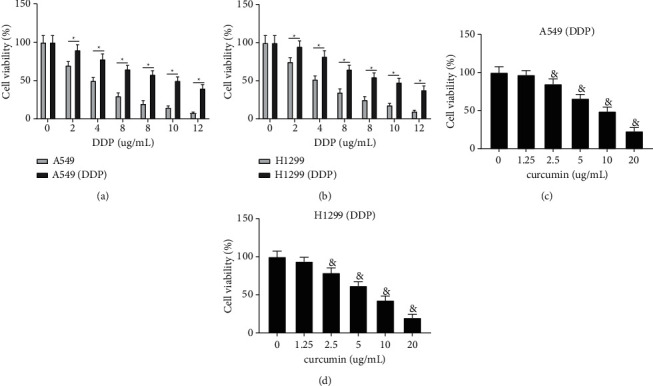
Curcumin can significantly decrease cell viability of drug-resistant NSCLC cells. (a, b) The effect of different concentrations of DDP on cell viability of A549, H1299, A549/DDP, and H1299/DDP cells. (c, d) The effect of curcumin on cell viability of A549/DDP and H1299/DDP cells. Experiment data are presented as the mean ± standard deviation (SD), each experiment was conducted 3 times. ^*∗*^*P* < 0.05, when compared with the A549/DDP or H1299/DDP group; ^&^*P* < 0.05, when compared with the 0 *μ*g/ml curcumin group. DDP, cisplatin; NSCLC, non-small-cell lung cancer.

The effect of various concentrations of DDP on A549, H1299, A549/DDP, and H1299/DDP cells was assessed by the CCK-8 assay. The results demonstrated that DDP can significantly decrease cell viability of A549 and H1299 cells in a concentration dependent manner, compared with those in A549/DDP and H1299/DDP cells (Figures 1(a) and 1(b), ^*∗*^*P* < 0.05). The medium inhibitory concentration (IC50) of DDP on A549 and H1299 cells was 4 *μ*g/mL, while that for A549/DDP and H1299/DDP cells was 12 *μ*g/mL, suggesting A549/DDP and H1299/DDP cells had obvious resistance to DDP, which was similar to the findings in a previous study [22]. Then, the cytotoxicity of curcumin against A549/DDP and H1299/DDP cells was assessed by the CCK-8 assay. As shown in Figures 1(c)and 1(d), curcumin can suppress the viability of A549/DDP and H1299/DDP cells in a concentration-dependent way. Specifically, cell viability of A549/DDP and H1299/DDP cells was reduced to approximately 48% after treatment with 10 *μ*g/mL curcumin for 48 h. Thus, the IC50 of curcumin against A549/DDP and H1299/DDP cells was 10 *μ*g/mL. In addition, the maximum nonlethal concentration of curcumin against A549/DDP and H1299/DDP cells was 2.5 *μ*g/mL. These results showed that curcumin can significantly decrease cell viability of drug-resistant NSCLC cells.

### 3.2. Curcumin Increases the Sensitivity of Resistant NSCLC Cells to DDP

**Figure 2:**
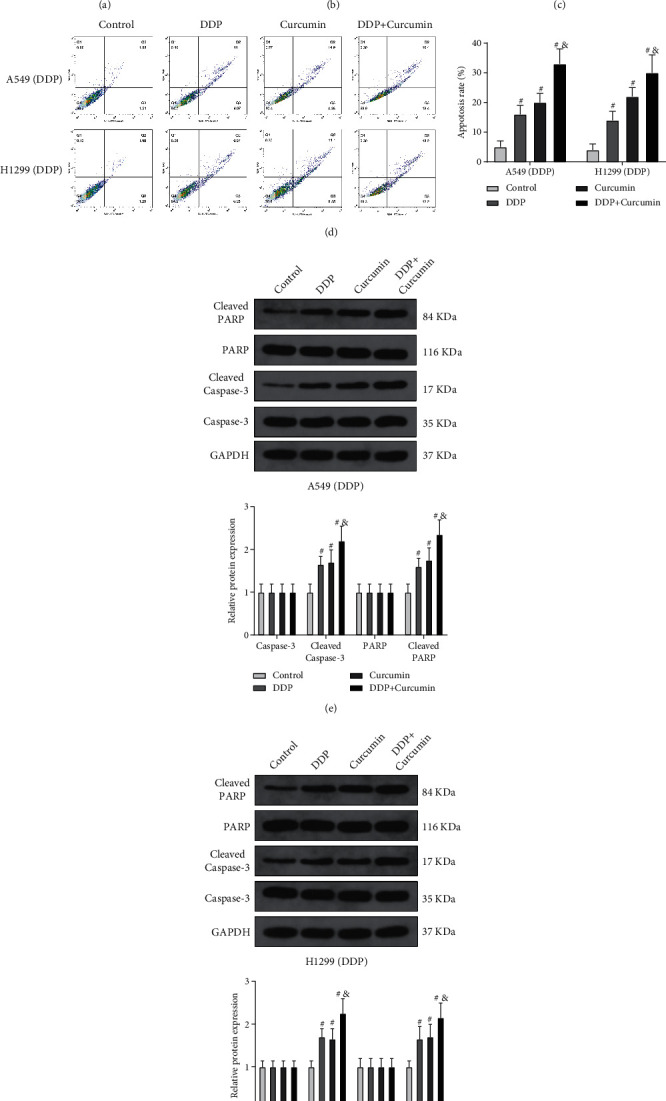
Curcumin increases the DDP sensitivity of resistant NSCLC cells. (a, b) The combined effect of different concentrations of DDP and curcumin (2.5 *μ*g/mL) on cell viability of A549/DDP and H1299/DDP cells. (c, d) After the cells were cotreated by DDP and curcumin (2.5 *μ*g/mL), cell viability and cell apoptosis were detected by CCK-8 and flow cytometry. (e, f) The expressions of cleaved caspase-3 and cleaved PARP were detected by western blotting. Experiment data are presented as the mean ± standard deviation (SD), each experiment was conducted 3 times. ^*∗*^*P* < 0.05, when compared with the DDP group; ^#^*P* < 0.05, when compared with the control group; ^&^*P* < 0.05, when compared with the DDP or curcumin group. DDP, cisplatin; NSCLC, non-small-cell lung cancer.

Although curcumin can suppress cell viability of A549/DDP and H1299/DDP cells, but whether curcumin can improve the sensitivity of A549/DDP and H1299/DDP cells to DDP remains unknown. We assessed the cell viability of A549/DDP and H1299/DDP cells after cotreatment by various concentrations of DDP and curcumin (2.5 *μ*g/mL). The CCK-8 assay showed compared with the DDP group that the cell viability of A549/DDP and H1299/DDP cells inthe DDP + curcumin group was significantly decreased (Figures 2(a) and 2(b), ^*∗*^*P* < 0.05), and the concentration of DDP for IC50 in the DDP + curcumin group was 2 *μ*g/mL. The combined effect of DDP (2 *μ*g/mL) and curcumin (2.5 *μ*g/mL) on A549/DDP and H1299/DDP cells was determined by the CCK-8 assay, which demonstrated that the cell viability of A549/DDP and H1299/DDP cells in the DDP + curcumin group was decreased significantly compared with the curcumin group and the DDP group (Figure 2(c), ^&^*P* < 0.05). Flow cytometry showed that the cell apoptosis rate in the curcumin group and the DDP group was elevated compared with that in the control group (^#^*P* < 0.05) but decreased when compared with that in the DDP + curcumin group. In addition to that, the promotive effect on cell apoptosis in the DDP + curcumin group was increased by 50% compared with that in the DDP group (Figure 2(d), ^&^*P* < 0.05). Measurement on expressions of apoptotic-related proteins (cleaved caspase-3 and cleaved PARP) by western blotting showed that the expressions of cleaved caspase-3 and cleaved PARP in the curcumin group and the DDP group were elevated compared with those in the control group (^#^*P* < 0.05) but decreased compared with those in the DDP + curcumin group (Figures 2(e)and2(f), ^#^*P* < 0.05). These results showed that curcumin can increase the sensitivity of resistant NSCLC cells to DDP, decrease cell viability, and promote cell apoptosis.

### 3.3. Curcumin Regulates the Expressions of ER Stress-Related Proteins

**Figure 3:**
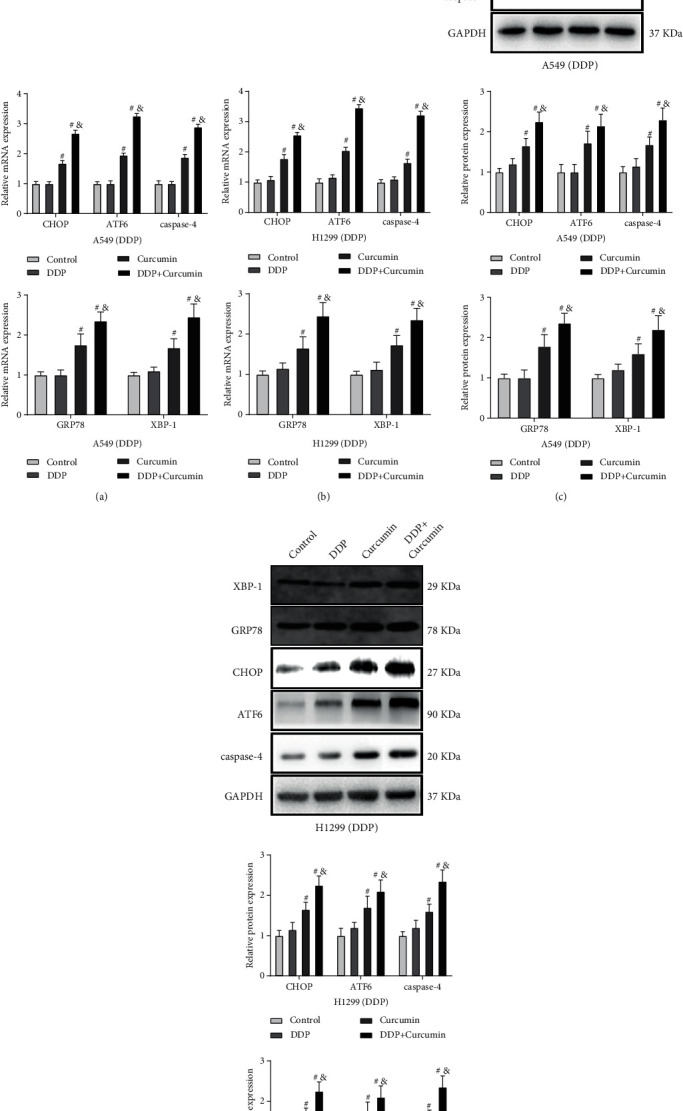
Curcumin can regulate the expressions of ER stress-related proteins in DDP-resistant NSCLC cells. (a–d) qPCR and western blotting detected the expressions of reticulum stress-related proteins, CHOP, GRP78, XBP-1, ATF6, and caspase-4; experiment data are presented as the mean ± standard deviation (SD), each experiment was conducted 3 times. ^#^*P* < 0.05, when compared with the control group; ^&^*P* < 0.05, when compared with the DDP or the curcumin group. DDP, cisplatin; NSCLC, non-small-cell lung cancer; and ER stress, endoplasmic reticulum stress.

Evidence in previous studies showed that ER stress is closely related to drug resistance of tumor cells [10, 23]. Therefore, we detected the expressions of ER stress-related proteins, including CHOP, GRP78, XBP-1, ATF6, and caspase-4 in A549/DDP and H1299/DDP cells. The expressions of ER stress-related proteins in the control group showed no significant difference with those in the curcumin group (Figures 3(a)–3(d)), but those expressions were elevated in the DDP group when compared with the control group (Figures 3(a)–3(d)). After the cells were cotreated by DDP and curcumin, the expressions of CHOP, GRP78, XBP-1, ATF6, and caspase-4 in the curcumin + DDP group increased significantly than those in the either curcumin or DDP group (Figures 3(a)–3(d)). These results showed that curcumin can regulate the expressions of ER stress-related proteins in DDP-resistant NSCLC cells.

### 3.4. Curcumin Increases DDP Sensitivity of Resistant NSCLC Cells through Regulating ER Stress

**Figure 4:**
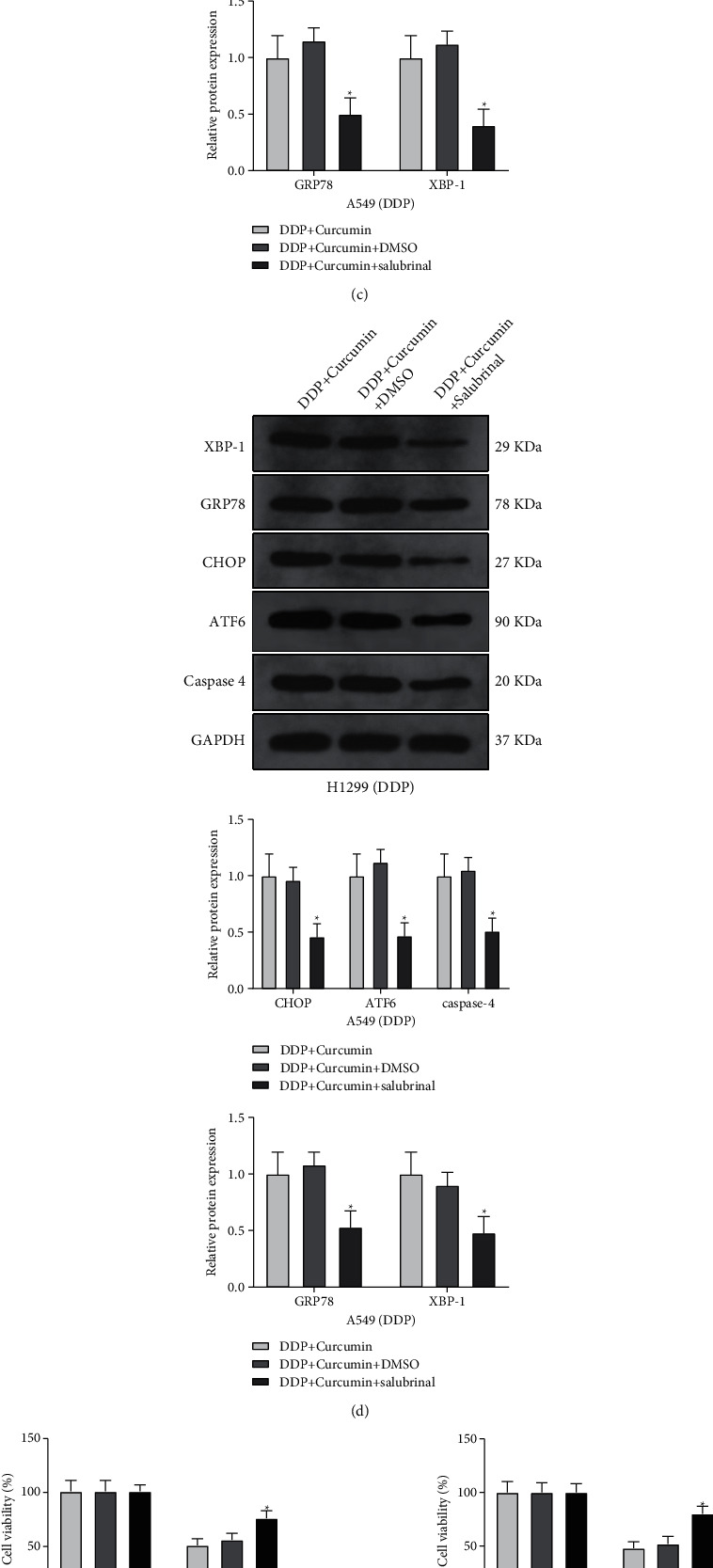
Curcumin increases the sensitivity to DDP-resistant NSCLC cells through regulating ER stress. After A549/DDP and H1299/DDP cells were cotreated with DDP and curcumin, salubrinal was used as an ER stress inhibitor to treat A549/DDP and H1299/DDP cells. (a–d) The expressions of CHOP, GRP78, XBP-1, ATF6, and caspase-4 were detected by qPCR and western blotting. (e) CCK-8 was used to detect cell viability; (f) the cell apoptosis rate was detected by flow cytometry. (g) Western blotting was used to measure the expressions of cleaved caspase-3 and cleaved PARP. Experiment data are presented as the mean ± standard deviation (SD), each experiment was conducted 3 times. ^*∗*^*P* < 0.05, when compared with the curcumin + DDP + DMSO group; DDP, cisplatin; NSCLC, non-small-cell lung cancer; ER stress, endoplasmic reticulum stress.

To determine whether curcumin can increase DDP sensitivity through regulating ER stress, we also treated the A549/DDP and H1299/DDP cells with ER stress inhibitor salubrinal (20 Μm) [24] with DMSO as control, after A549/DDP and H1299/DDP cells were cotreated with DDP and curcumin. Detection on ER stress-related proteins showed that compared with the curcumin + DDP + DMSO group, the expressions of CHOP, GRP78, XBP-1, ATF6, and caspase-4 were decreased significantly in both A549/DDP and H1299/DDP cells (Figures 4(a)–4(d)). Subsequently, we measured the effect of salubrinal on the cell viability and apoptosis rate. CCK-8 and flow cytometry showed that compared with cells in the curcumin + DDP + DMSO group, A549/DDP and H1299/DDP cells in the curcumin + DDP + salubrinal group had elevated cell viability and decreased the apoptosis rate (Figures 4(e)and4(f)). Western blotting also demonstrated that the expressions of cleaved caspase-3 and cleaved PARP in the curcumin + DDP + salubrinal group were suppressed compared with those in the curcumin + DDP + DMSO group (Figure 4(g)).

The working mechanism of curcumin increasing DDP sensitivity of resistant NSCLC cells through ER stress. DDP, cisplatin; NSCLC, non-small-cell lung cancer; and ER stress, endoplasmic reticulum stress.

## 4. Discussion

DDP is considered to be one of the most promising chemotherapy drugs broadly used for various types of human epithelial cancers, including ovarian carcinoma, lung carcinoma, breast carcinoma, and head and neck carcinoma [25–27]. Nevertheless, DDP resistance is a major challenge for cisplatin-based chemotherapy. Cisplatin-resistant cells exhibit decreased intracellular DDP accumulation due to enhanced efflux and reduced influx [28]. DDP can also be inactivated by sulfur-containing macromolecules, including metallothionein and glutathione [29]. Exploration of novel strategies to increase the sensitivity of cancer cell to DDP for overcoming the drug resistance will be highly desirable. In this study, we demonstrated that the exposure of cancer cells to sublethal doses of curcumin could promote DDP chemotherapeutic efficiency against NSCLC cells, in which the ER stress pathway mediated by the CHOP, ATF6, and caspase-4 plays an important role.

Curcumin, as a cancer chemosensitizing agent, can effectively reduce resistance to many chemotherapy drugs, including DDP, mitomycin C, and paclitaxel, in a wide variety of tumor cells [7, 8, 10, 15, 16]. Due to its chemosensitizing effect, a combination of cisplatin with curcumin was proposed to improve the sensitivity of DDP. Zhang et al. stated curcumin enhances DDP sensitivity of human NSCLC cell lines through influencing a Cu-Sp1-CTR1 regulatory loop [8]. Recently, Ye et al. reported curcumin reverses DDP resistance and promotes human lung adenocarcinoma A549/DDP cell apoptosis through HIF-1*α* and caspase-3 mechanisms [15]. In our study, we first found that curcumin inhibits the proliferation of DDP-resistant A549/DDP and H1299/DDP cells in a dose-dependent manner, as well as in DDP sensitive A549 and H1299 cells. These results are in agreement with previous studies, showing that curcumin induces apoptosis and inhibits the proliferation of cancer cells. Similar antitumor effects can also be found in other plant extracts [30, 31]. In addition, the DDP-chemosensitizing effects of curcumin against NSCLC cell viability and apoptosis were demonstrated by using the CCK-8 assay and the flow cytometer.

ER stress causes apoptosis through following mechanisms, including expression of CHOP, activation of ER resident caspase, and induction of the ASK1-JNK pathway [32]. In particular, CHOP is the first molecule identified to mediate ER stress-induced apoptosis. The unfolding protein response of ER stress exceeds the threshold, and damaged cells are committed to apoptosis through the ATF6-mediated GADD153 signaling pathway [33]. Recently, ER stress is one of the molecular mechanisms responsible for curcumin-induced apoptosis [34, 35]. In this work, to validate the hypothesis that curcumin increased the sensitivity of NSCLC to DDP through the ER stress pathway, we detected the expression levels of ER stress-related proteins (CHOP, ATF6, and caspase-4). As expected, combined treatment of curcumin and cisplatin significantly increased protein and RNA levels of CHOP, ATF6, and caspase-4 in comparison with curcumin or cisplatin alone. In addition to that, the combination of curcumin and DDP on A549/DDP and H1299/DDP cells led to the decreased cell viability and elevated apoptosis rate, indicating curcumin increased DDP sensitivity through regulating ER stress. This result was further supported by the elevated cell viability and decreased apoptosis rate in A549/DDP and H1299/DDP cells treated with combination of curcumin, DDP, and ER stress inhibitor salubrinal.

In conclusion, the current study revealed that the ER stress pathway is associated with acquired DDP resistance in NSCLC cells and curcumin can enhance the chemosensitizing effect of NSCLC cells by targeting the ER stress pathway. Taken together, this study demonstrated curcumin can increase DDP sensitivity of NSCLC cells through mediating the ER stress pathway. A better understanding on the drug resistance of cancer cell can facilitate the solution against drug resistance. The results of this study proposed a possible mechanism of curcumin improving DDP sensitivity in NSCLC cells and may shed a little light on developing the molecular targets in the ER stress pathway to overcome the DDP resistance in NSCLC.

## Figures and Tables

**Table 1:** Primer sequences for the real-time quantitative PCR assay.

Genes	Sequences (5′ end to 3′ end)
CHOP-F	ATGAATCTGCACCAAGCATGA
CHOP-R	CAGGTGGGTAGTGTGGCCC
Caspase-4-F	GGGAGAAGGACTTCATTG
Caspase-4-R	TAAGCATGTGATGAGTTG
ATF6-F	AGCTCCATGCTTAAGGAC
ATF6-R	GGGATAGGTGAT GATGAA
GRP78-F	AAGCCCGTCCAGAAAGTGTT
GRP78-R	ATCTGGGTTTATGCCACGGG
XBP-1-F	GGGACCCCTAAAGTTCTGCT
XBP-1-R	CCACTTGCTGTTCCAGCTCA
GAPDH-F	GGTGAAGGTCGGAGTCAACG
GAPDH-R	TGAAGGGGTCATTGATGGCAAC

F, forward; R, reverse.

## Data Availability

The authors can make data available on request through a data access committee and institutional review board. In addition, all the data can also be obtained from author Lile Wang.
